# Map of Cuba

**Published:** 1898-04

**Authors:** 


### BOOK NOTICES.

Map of Cuba. This is a beautiful colored map, mounted on heavy white paper and having all the details up to date. Its size is thirty-four inches by seventeen and a half 34x17½). It is issued by the Delaware Fire Insurance Co. of Philadelphia to its patrons free of charge. To those who are not its patrons the map will be sent for the nominal charge of seventy-five cents.

